# Fabrication and characterization of an antibacterial chitosan/silk fibroin electrospun nanofiber loaded with a cationic peptide for wound-dressing application

**DOI:** 10.1007/s10856-021-06542-6

**Published:** 2021-08-28

**Authors:** Sadjad Khosravimelal, Milad Chizari, Behrouz Farhadihosseinabadi, Mehrdad Moosazadeh Moghaddam, Mazaher Gholipourmalekabadi

**Affiliations:** 1 Applied Biotechnology Research Center, Baqiyatallah University of Medical Sciences, Tehran, Iran; 2 Hematopoietic Stem Cell Research Center, Shahid Beheshti University of Medical Sciences, Tehran, Iran; 3 Department of Tissue Engineering & Regenerative Medicine, Faculty of Advanced Technologies in Medicine, Iran University of Medical Sciences, Tehran, Iran

## Abstract

Wound infections are still problematic in many cases and demand new alternatives for current treatment strategies. In recent years, biomaterials-based wound dressings have received much attention due to their potentials and many studies have been performed based on them. Accordingly, in this study, we fabricated and optimized an antibacterial chitosan/silk fibroin (CS/SF) electrospun nanofiber bilayer containing different concentrations of a cationic antimicrobial peptide (AMP) for wound dressing applications. The fabricated CS/SF nanofiber was fully characterized and compared to the electrospun silk fibroin and electrospun chitosan alone in vitro. Then, the release rate of different concentrations of peptide (16, 32, and 64 µg/ml) from peptide-loaded CS/SF nanofiber was investigated. Finally, based on cytotoxic activity, the antibacterial activity of scaffolds containing 16 and 32 µg/ml of the peptide was evaluated against standard and multi-drug resistant strains of *Staphylococcus aureus*, *Escherichia coli*, and *Pseudomonas aeruginosa* isolated from burn patients. The peptide-loaded CS/SF nanofiber displayed appropriate mechanical properties, high water uptake, suitable biodegradation rate, a controlled release without cytotoxicity on Hu02 human foreskin fibroblast cells at the 16 and 32 µg/ml concentrations of peptide. The optimized CS/SF containing 32 μg/ml peptide showed strong antibacterial activity against all experimental strains from standard to resistance. The results showed that the fabricated antimicrobial nanofiber has the potential to be applied as a wound dressing for infected wound healing, although further studies are needed in vivo.

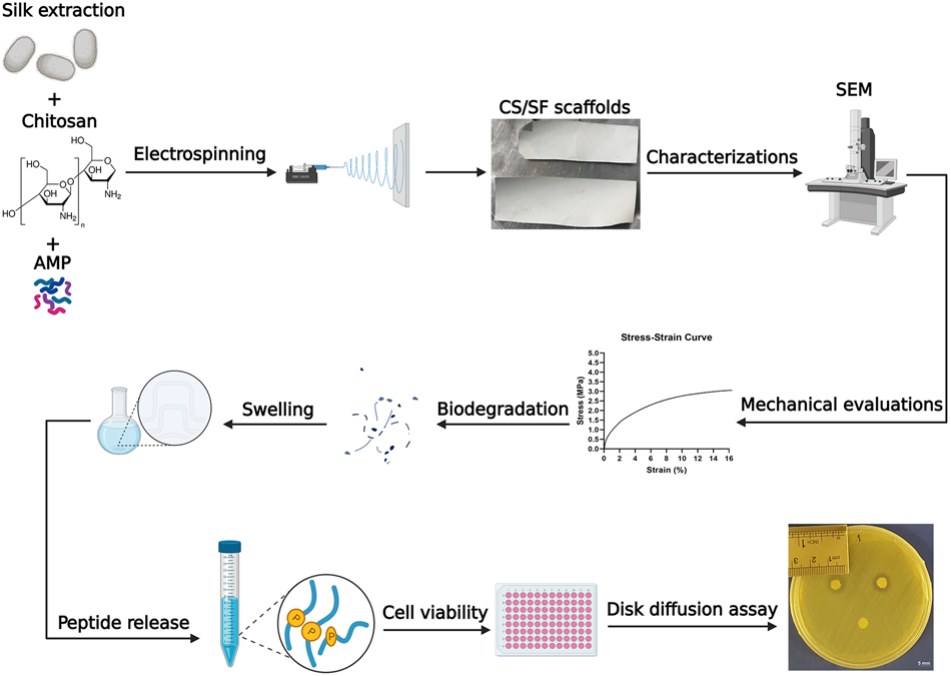

## Introduction

Wound infections are among the most common infections, especially burns. Predominantly, antibiotic therapy along with using antiseptics like povidone-iodine in the wound bed is the most common treatment option for this type of infection. However, in some severe cases, hospitalization is required [1, 2]. In these cases, the infection causes a prolonged inflammation activity and thus an extended healing period [3]. Unfortunately, in recent years, the remarkable emergence of bacteria that are resistant to antibiotics used to treat these types of infections has jeopardized the effective treatment of such wounds [4]. Therefore, it is necessary to use new strategies that accelerate the wound healing process by eliminating this type of infection. In the presented study, we have designed and developed an antimicrobial peptide (AMP)-loaded bilayer chitosan and silk fibroin (AMP-loaded CS/SF) electrospun nanofiber as a dressing for infected wounds and soft tissues applications.

Biomaterials-based wound dressings are now a choice to cover the wound site and facilitate the healing process. Ideally, a dressing should have mechanical properties enough to protect the wound bed, maintain homeostasis, be cytocompatible, and display antibacterial activity [5, 6]. Although the innate antibacterial properties can be a barrier to pathogen invasion, however, the addition of an antimicrobial agent to the dressing is now attracting more interest, especially with the increase of resistant bacteria [7]. In recent years, a significant number of studies have been performed on the fabrication of scaffolds with antimicrobial properties based on several strategies to produce wound dressings such as hydrogels [8] and electrospun membranes [7]. Among them, electrospun fibers have been used in biomedical applications in a wide range as they can act like a natural extracellular matrix for cell motility and propagation. In addition, they provide a large surface-to-volume ratio due to their porous structure [9, 10]. Natural biomaterials have been extensively applied for electrospinning purposes to be utilized as wound dressings due to biocompatibility reasons [11].

Chitosan (CS) is a natural polysaccharide derived from chitin that has some distinctive properties appropriate for biomedical purposes [12]. CS has shown biocompatibility, desired biodegradability, some degrees of antibacterial activities, and also is inexpensive which makes it a favorable option for tissue engineering and regenerative medicine applications [13, 14]. However, due to the mechanical weakness of using CS alone, its application is decreased [15]. Regarding this issue, it has been proposed to use CS in combination with some other mechanically strong materials [16]. Hence, its combination with silk fibroin (SF) can be one of the strategies. SF is the major protein of *Bombyx mori* silkworm cocoons that is famous for its toughness and elasticity. Additionally, due to its biocompatibility and easy handwork, it has been widely used in clinical studies in a wide range and some of its products have been approved by the FDA [17]. Previously, the successful electrospinning of the SF on the decellularized amniotic membrane surface has been reported for improving its mechanical properties [18]. Accordingly, we hypothesized to overcome the mechanical weakness of CS by the same strategy. Also, such electrospun mats have been used in combination with some antimicrobial agents as an antibacterial wound dressing [7].

Cationic AMPs, short peptides found in the immune system of many species, have recently received much attention as one of the most important alternatives to conventional antibiotics [19, 20]. In general, they act directly against a wide range of pathogens such as bacteria, viruses, and fungi. Some of the AMPs are now commercially available [21, 22]. CM11 is a short cecropin-melittin-derived cationic AMP that is comprised of 11 amino acid residues (WKLFKKILKVL). This peptide has been shown in some studies to have antibacterial activity against many Gram-positive and -negative antibiotic-resistant pathogens. [23, 24]. Therefore, its potential for use in biomaterial-based wound dressings can be considered.

In the current study, an antibacterial CS/SF electrospun nanofiber containing different concentrations of CM11 peptide was fabricated and evaluated in vitro. For this purpose, the mechanical properties, biocompatibility, characterizations, and drug release profile of AMP-loaded CS–SF were examined. Eventually, the antibacterial activity of AMP-loaded CS-SF nanofiber was evaluated against standard and multi-drug resistant (MDR) strains of *Escherichia coli*, *Staphylococcus aureus*, and *Pseudomonas aeruginosa* isolated from burn patients in vitro. This optimized antibacterial wound dressing is introduced for future in vivo and clinical investigations of tissue engineering and regenerative medicine applications.

## Materials and methods

### Bilayer scaffold fabrication

#### Silk fibroin extraction and characterization

Fresh *Bombyx mori* silkworm cocoons were supplied from the Iranian Silkworm Research Center (Rasht, Iran). SF was extracted according to the method described by Gholipourmalekabadi et al. [25]. Briefly, cocoons were dissected and boiled in 0.02 M Na_2_CO_3_ solution for 1 h. To remove the glue-like protein (Sericin), cocoons were completely rinsed with distilled water in a process called degumming. Then, for 4 h at 60 °C, the degummed SF was dissolved in 9.3 M Lithium Bromide (LiBr). Afterward, it was dialyzed against ultrapure water for 72 h (MWCO of 12 KDa). The final solution was lyophilized using a freeze-dryer apparatus (Nanbei, Zhengzhou, China) for 24 h and then restored at room temperature for further applications.

#### Chitosan preparation

To prepare CS solution for electrospinning, the polyvinyl alcohol (PVA, Sigma-Aldrich, St. Louis, MO, USA) was added to deionized water to form a 12% w/v solution and stirred for 4 h at 80 °C (350 rpm). Next, the medium molecular weight CS (Sigma-Aldrich, St. Louis, MO, USA) was dissolved in 2% acetic acid (Merck, Darmstadt, Germany) to prepare a 3% w/v solution and stirred on a magnetic stirrer for 15 h. Afterward, this solution was added to PVA and gently stirred for an additional 2 h. Then, it was stored at 4 °C for subsequent steps.

#### Fabrication of antibacterial bilayer mat by direct electrospinning

The bilayer mat was fabricated by direct electrospinning of AMP-loaded CS on ethanol-treated SF. For the electrospinning process, the lyophilized SF was dissolved in 98% formic acid (Sigma-Aldrich, USA) to form a 10% solution. The electrospinning was performed on an aluminum foil-covered collector for 6 h under the following conditions as described elsewhere [25]: a 2-ml syringe (gauge 23), 18 kV/cm of voltage, 0.3 ml/h flow rate, and a 150 mm distance. Eventually, SF was immersed in 70% ethanol to induce β-sheet formation.

CM11 peptide (Biomatik, Canada) was added to prepared CS solution in 16, 32, and 64 µg/ml concentrations that are based on our previous study [26]. According to these studies, the MIC of peptide in the culture medium was 8 µg/ml. To obtain a 50/50% v/v bilayer, the solution was electrospun on the SF fibers for another 9 h in a 2-ml syringe (gauge 23) under electrospinning conditions of a 20 kV voltage, 0.2 ml/h of flow rate, and a distance of 200 mm as described by De Vrieze et al. [27] with some alterations. The bilayer was crosslinked by glutaraldehyde (GTA) vapor at the final stage.

### Scaffold characterizations

#### Silk fibroin characterization by FTIR

To determine the functional groups of the extracted silk fibroin protein, the lyophilized SF was mixed with potassium bromide (KBr). The formed pellets were analyzed by Fourier transform infrared spectroscopy (FTIR) using 55FTIR EQUINOX spectrophotometer (BrukerOptik GmbH, Germany) in the range of 4000–400 cm^−1^ with a 4 cm^−1^ of resolution.

#### Morphology of electrospun fibers

The fabricated CS fibers and ethanol-treated SF scaffolds were gold-sputtered to be observed under the scanning electron microscope (SEM, AIS2100; Seron Technology, Uiwang-si, Gyeonggi-do, South Korea) at an acceleration voltage of 15 kV. The uniform structure of the scaffolds and the formation of fibers were expected to be observed without the presence of any beads.

#### Mechanical evaluations

All fabricated scaffolds were cut into rectangular pieces (4 cm × 1 cm). Using a microtome (Thermo Fisher Scientific, Waltham, Massachusetts, USA) slices of 5 μm in thickness were prepared for all samples to analyze and compare their mechanical characteristics under a universal tensile machine (Santam, Tehran, Iran). Using a 50 N load cell, the test was performed at a strain rate of 10 mm/min (*n* = 5). The results were obtained from the stress–strain curve. The mean of each measurement was reported.

#### In vitro biodegradation

The biodegradation of the scaffolds was calculated by embedding 70 ± 7 mg of all samples (*M*_0_) into 10 ml of simulated body fluid (SBF) supplemented with a 0.1 wt% enzyme (Merck, Darmstadt, Germany) and then, incubated at 37 °C. After incubation, three samples were removed from SBF at each predetermined time interval, washed with deionized water, and dried for 72 h at 25 °C. Afterward, the samples were weighed again (*M*_d_), and according to the following equation the percentage of the lost weight was measured:

1 $${\mathrm{Weight}}\,{\mathrm{loss}}\left( \% \right) = \frac{{{\mathrm{M}}0 - {\mathrm{Md}}}}{{{\mathrm{M}}0}} \times 100$$

#### Swelling rate

To determine the swelling rate of the bilayer scaffolds, dried scaffolds were weighed (*W*_d_) and then, immersed in 5 ml of deionized water for 24 h at 25 °C. Afterward, the scaffolds were removed from the water at each predetermined time interval and their wet weights were measured and denoted by *W*_s_. The swelling rate of the scaffolds was calculated according to the following formula:

2 $${\mathrm{Swelling}}\,{\mathrm{rate}}\left( \% \right) = \left( {\left[ {{\mathrm{W}}_{\mathrm{s}}--{\mathrm{W}}_{\mathrm{d}}} \right]/{\mathrm{W}}_{\mathrm{d}}} \right)$$

#### In vitro peptide release

To evaluate the release of CM11 peptide from the scaffolds, they were submerged in PBS at 37 °C for eight consecutive days. After each time interval, the optical density of the samples was measured at 280 nm using NanoDrop One microvolume UV–Vis Spectrophotometers (Thermo Fisher Scientific, Waltham, Massachusetts, United States). The results were the mean of three different examinations.

### Biocompatibility assay

#### Cell viability

For cell viability assessment, the MTT assay was performed. In brief, the scaffolds were seeded by Hu02 human foreskin fibroblast cells obtained from the Iranian Biological Resource Center (Tehran, Iran) for 1, 3, and 7 days at 37 °C under standard conditions (95% humidity and 5% CO_2_). After each time interval, the samples were treated by 3-(4,5-Dimethyl-2-thiazolyl)-2,5-diphenyl-2H-tetrazolium bromide (MTT) solution for 4 h at 37 °C in dark. Afterward, the formed tetrazolium crystals were dissolved in Dimethyl sulfoxide (DMSO) for 15 minutes in dark. This method was repeated thrice and all the results were compared to control (cells cultured in cell culture plate) using a plate-reader (DANA, DA3200) at 570 nm. The viability of the cells grown on the cell culture plate (control sample) was considered 100%. The scaffolds showing some degrees of toxicity were excluded from further assessments.

#### Scaffold–cell interaction

The cells were seeded on the scaffolds and incubated for 3 days at 37 °C under standard conditions (95% humidity and 5 % CO_2_). For taking images under SEM, the cell-seeded scaffolds were fixed with 2.5% glutaraldehyde (Merck, Darmstadt, Germany) for 2 h. The samples were then submerged in phosphate-buffered saline (PBS) at 25 °C for 30 min. afterward, they were dehydrated by a graded series (30, 50, 70, and 100%) of ethanol, and followed by drying under vacuum for 12 h. After gold sputtering, the scaffolds were observed under SEM.

### Antibacterial activity

Based on MTT results, the antibacterial activity of the CS/SF, Peptide-loaded CS/SF (16 µg/ml) and Peptide-loaded CS/SF (32 µg/ml) scaffolds was evaluated against standard strains of *Staphylococcus aureus* (ATCC 25923), *Escherichia coli* (ATCC 25922), *Pseudomonas aeruginosa* (ATCC 28753), and their MDR strains (MDR 50, all) isolated from burn patients by disk diffusion assay according to clinical and laboratory standards institute guidelines [28]. Briefly, 100 µl of 0.5 McFarland (1.5 × 10^8^ CFU/ml) bacterial suspensions were cultured on Mueller-Hinton Agar (MHA) plates. Then, the prepared disks of the samples (0.5 cm in diameter) were placed on the center of plates, and were incubated at 37 °C for 24 h. The inhibition zone of bacterial growth was reported using a ruler and compared among samples. All human sample collections were approved by the Baqiyatallah University of Medical Sciences ethical committee under approval ID of IR.BMSU.REC.1398.361.

### Statistical analysis

All the experiments were conducted in triplicates or with at least three different samples and the mean of each was recorded. The collected data were analyzed using GraphPad Prism v8.0.2.263 software. Where appropriate, independent samples *t* test or One-way ANOVA were applied. The data were expressed as mean ± SD. In addition, *P* < 0.05 was considered as the level of significance.

## Results

### Scaffold characterizations

#### Silk fibroin characterization by FTIR

**Fig. 1:**
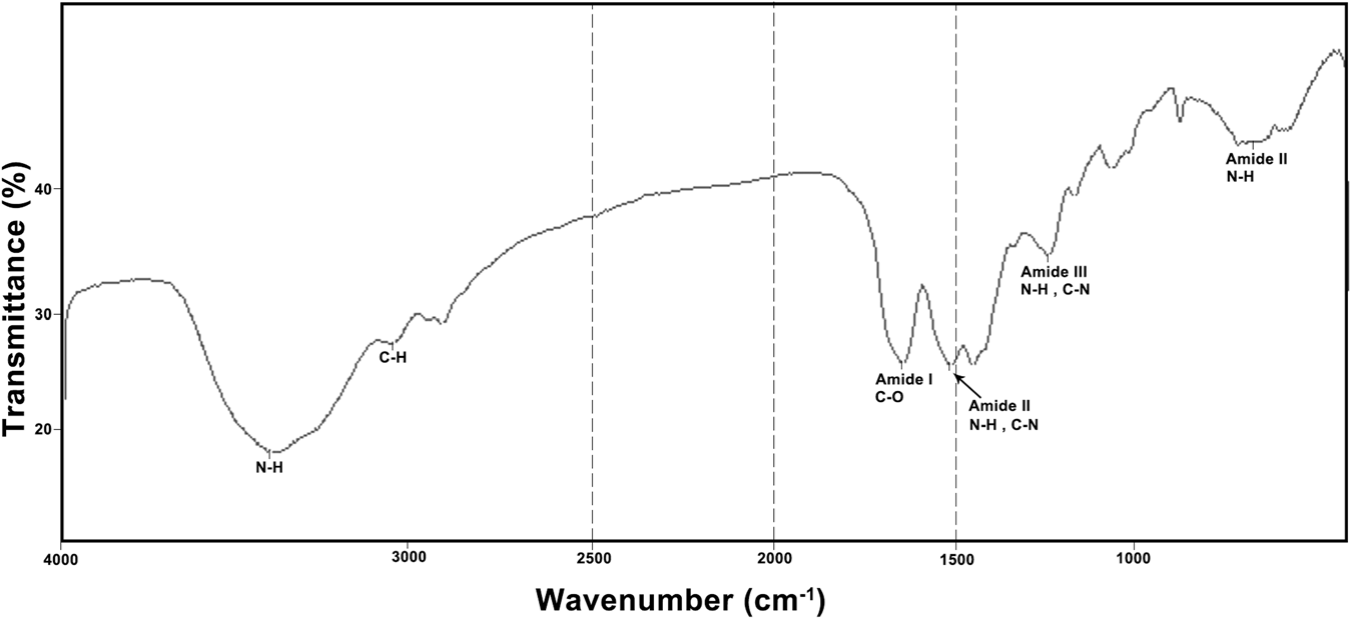
FTIR spectra of silk fibroin. FTIR spectra of silk fibroin.The bands at 1648 cm^−1^, 1513 cm^−1^, and 1241 cm^−1^ represent amide I (C=O stretching), amide II (secondary N-H bending) and amid III (C–N and N–H functionalities) groups, respectively

FTIR was performed to confirm the molecular composition of the extracted SF protein before ethanol treatment. As shown in Fig. 1, proteins display characteristic vibration peaks at 1650–1630 cm^−1^, 1540–1520 cm^−1^, and 1270–1230 cm^−1^ for Amide I, amide II, and amide III, respectively. These peaks are distinguishable vibration peaks for all protein samples.

#### Morphology electrospun fibers

**Fig. 2:**
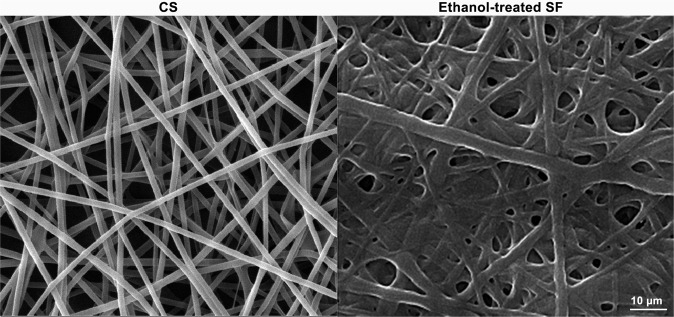
The ultrastructure of the electrospun CS and electrospun SF after treating with ethanol observed under SEM

The morphology of the electrospun CS and SF (after ethanol treatment) scaffolds under SEM is presented in Fig. 2. The uniform structure of the fibers without bead formation is distinctly evident in both scaffolds. However, ethanol induces the β-sheet formation to the SF fibers and thus improves the biomechanical properties of the final product.

#### Mechanical evaluations

Mechanical properties are essential determinants of a biomaterial appropriate to be used in tissue engineering. The results obtained from the stress–strain curve are summarized in Table 1. As expected, electrospinning of SF has improved the mechanical properties of CS. It clearly can be apprehended from the table that there is a considerable difference between the electrospun CS/SF bilayer with electrospun CS. The bilayer displays mechanical characteristics similar to electrospun SF. As SF is one of the toughest known biomaterials, these results are considerable.

**Table 1:** The biomechanical properties of the electrospun SF, CS, and SF/CTS scaffolds

Material	SF	CS	CS/SF
Ultimate tensile strength [MPa]	4.33 ± 0.04	3.89 ± 0.29	4.15 ± 0.19
Elongation at break (%)	10.9 ± 0.05	6.9 ± 0.45	10.4 ± 0.73

#### In vitro biodegradation

**Fig. 3:**
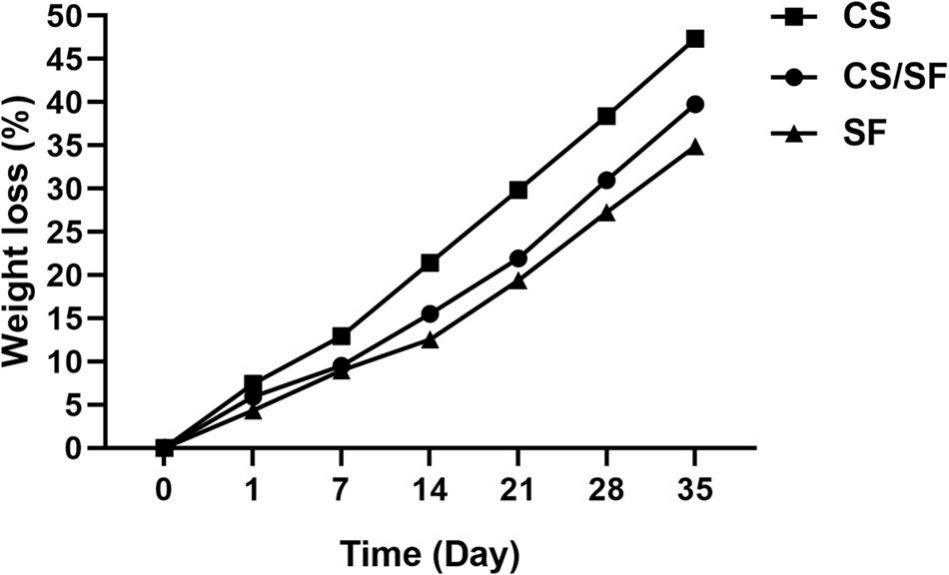
The biodegradation rate of the scaffolds after 35 days. CS significantly degrades faster from day 1 to 35 compared to other samples in all time intervals (*P* < 0.05). There are no significant differences between SF and CS/SF scaffolds during 21 days (*P* > 0.05). CS/SF sample degrades significantly faster than SF alone from day 21 to 35 in all-time intervals (*P* < 0.05)

Figure 3, shows the biodegradation rate of the samples in a time period of 35 days in enzyme-supplemented SFB. CS degrades faster and losses almost half of its initial weight within 35 days. SF and CS/SF experimental samples follow approximately a similar manner in 3 weeks. However, from day 21 to 35, CS/SF scaffold shows more biodegradation compared to SF. The differences among samples containing SF with chitosan are significant (*P* < 0.05). After 35 days, the bilayer lost almost 40% of its dry weight. The results emphasize the effect of SF on the desirable degradation of CS in the bilayer.

#### Swelling rate

**Fig. 4:**
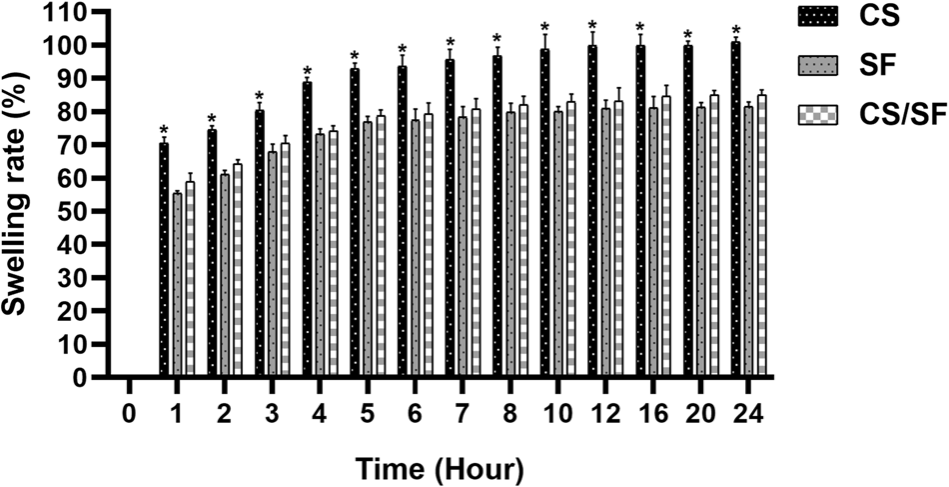
The swelling rate of scaffolds. The figure shows a similar swelling pattern among all samples within 24 h. However, CS shows a significant water uptake in all time intervals compared with SF and CS/SF samples (*P* < 0.0001). There are no considerable differences between SF and CS/SF samples in all predetermined time intervals. * represents a significant difference with SF and CS/SF (*P* < 0.0001)

The water absorption examination also revealed a significant difference between SF and CS/SF scaffolds with CS (*P* < 0.05). According to Fig. 4, a dramatic water uptake after 4 h and a slight increase between 5 and 10 h is observable among all three samples. However, the CS sample absorbs more water than FS and CS/SF. After the first 10 h, a constant level of water absorption is represented up to 24 h in all experimental samples. Although SF and CS/SF samples display high water uptake, the swelling rate of CS is around twofold after 24 h.

#### In vitro peptide release

**Fig. 5:**
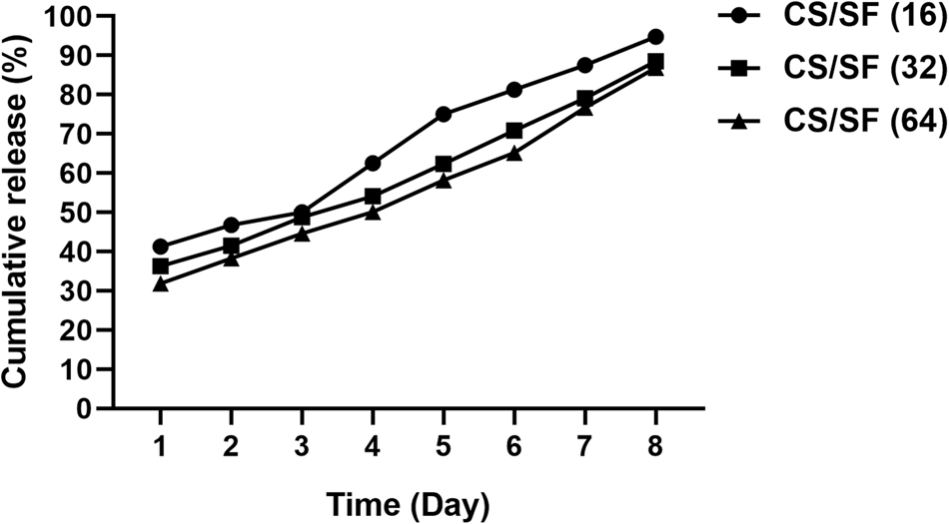
The release pattern of CM11 peptide from the scaffolds. A burst release of the peptide at day 1, and an increase in a linear manner until day 8 is evident in the figure in all samples. Approximately 90% of the peptide is released through 8 consecutive days from all three experimental samples

Peptide-loaded SF/CTS samples containing 16, 32, and 64 µg/ml peptide, respectively, were considered to study their release behavior in vitro. According to the results represented in Fig. 5, a burst release of CM11 peptide from scaffolds is recorded after day 1 (more than 30% in all samples). An increase in the release of peptides from the bilayer in a linear manner is observable up to 8 days, as all scaffolds released almost 90% of their peptide until this day. All three samples display almost a similar pattern with different rates.

### Biocompatibility assay

Cell viability and attachment assays are performed to show the compatibility of the products to native residential cells.

#### Cell viability

**Fig. 6:**
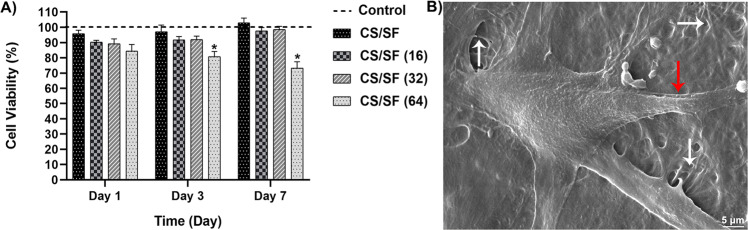
**A** Cell viability results of the 4 investigated bilayer scaffolds with different concentrations of the peptide, show a significant difference in fibroblasts’ growth of peptide-loaded CS/SF (64 µg/ml peptide) scaffold and the control in days 3 and 7 post-seeding. **B** adhesion of a fibroblast cell to the surface of the bilayer fibers. White and red arrows represent the fibers and cell filopodia, respectively

The fabricated bilayer scaffold and bilayer scaffolds loaded with different concentrations of CM11 peptide were investigated by MTT assay in 1, 3, and 7 days and compared with control (seeded cells without scaffolds) to check if they induce cytotoxicity. As demonstrated in Fig. 6A, there were no significant differences between the under-examination scaffolds with control on day 1 post-seeding. However, on days 3 and 7, a significant decrease in Hu02 fibroblast cells’ growth was observed in the peptide-loaded SF/CTS scaffold (64 µg/ml peptide) compared with the control. Meantime, no considerable differences with control were recorded in 3 other samples in any predetermined time interval. As the results, the peptide-loaded SF/CTS scaffold containing 64 µg/ml peptide was excluded from the study, and the other groups were considered for further evaluation.

#### Cell attachment

The adhesion of cells to the scaffolds is a direct representative of the cell–scaffold adaptation. Here in Fig. 6B, the morphology of a Hu02 fibroblast cell well attached to the surface of the fibers is clearly observable after 3 days of culturing on the bilayer.

### Antibacterial activity

**Fig. 7:**
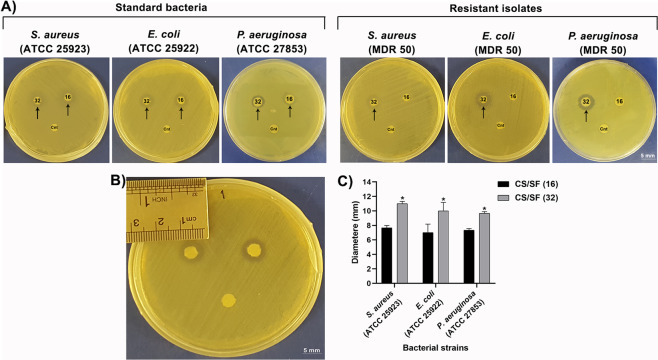
Assessment of antibacterial activity peptide-loaded CS/FS by disk diffusion assay. **A** The antibacterial activity peptide-loaded CS/SF (16 & 32 µg/ml peptide, respectively) bilayers are compared with control (Cnt). A halo around peptide-loaded bilayers is visible after 24 h in standard strains of *S. aureus, E. coli*, and *P. aeruginosa* MHA plates compared to control disks. In resistant strains, an inhibition zone is observable only around CS/SF containing 32 µg/ml CM11 peptide. **B** The diameter of the growth inhibition zone recorded by a ruler. **C** Diameters around peptide-loaded CS/SF (16 µg/ml) disks are compared to peptide-loaded CS/SF (32 µg/ml). There are significant differences in the scaffolds containing higher concentrations of CM11 peptide (32 µg/ml) with peptide-loaded CS/SF containing 16 µg/ml peptide in both standard strains (*P* < 0.05). Results from resistant strains and all controls are excluded as no diameters have formed and there is nothing to compare

The results taken from the disk diffusion assay are presented in Fig. 7. CS/SF and peptide-loaded CS/SF (16 and 32 µg/ml peptide, respectively) disks were placed in MHA plates cultured with standard and resistant strains of *S. aureus, E. coli*, and *P. aeruginosa*, and incubated for 24 h. According to the results (Fig. 7A), the CS/SF scaffold shows no antibacterial activity at all, while in peptide-loaded CS/SF (16 µg/ml) scaffold, a bacterial growth inhibition zone is evident against standard strains. This scaffold has no antibacterial effects on resistant bacteria isolated from burn patients. Meanwhile, a zone of growth inhibition against all standard and resistant strains are revealed around disks of peptide-loaded CS/SF (32 µg/ml) scaffold. Figure 7B demonstrates the size of the halo around an experimental disk, while the analysis of the recorded diameters is presented in Fig. 7C to quantify the results taken from the disk diffusion assay.

## Discussion

This study was presented to inquire about the ability of an AMP-loaded CS/SF electrospun bilayer mat as a possible scaffold for wound infection applications. In recent years, antimicrobial peptides have been considered as a promising option for the post-antibiotic era in many studies [29–32]. These peptides are important members of the host defense system in eukaryotes that have a broad function in wound healing, immune regulation, and inflammation, and act as endogenous antibiotics [33]. Due to their antimicrobial and biological potentials, these peptides have also been considered for wound dressing purposes. [8, 34, 35]. Accordingly, many studies have been done to use these peptides against infections caused by antibiotic-resistant bacterial strains. However, these peptides may have cytotoxic effects, so their controlled-release can be effective in reducing cytotoxicity. CM11 is a synthetic hybrid of cecropin-melittin that has shown stronger antibacterial activities than both of its compartments with less hemolytic and cytotoxic effects [36]. Studies have shown that the antimicrobial activity of this peptide may be associated with its ability to cell membrane infiltration and disruption via the formation of pores in cell membranes [37]. Here, we designed and fabricated an electrospun bilayer of SF and CS scaffold for controlled-delivery of CM11 peptide and examined its potential as a dressing for infected wounds with the determination of its antimicrobial activity against MDR strains of Gram-negative and -positive bacteria especially *P. aeruginosa* as the most common Gram-negative strain in burn patients.

Electrospinning of polyelectrolyte materials that possess electric charges along a chain like CS is difficult as they display greater shear thinning than neutrals [38]. As the result, CS has been electrospun in blended solutions with other electrospinnable materials such as PVA, that can interact with CS through hydrogen bonding [39]. Despite a uniform structure, the fibers formed from the blended solution of CS/PVA are mechanically weak. Thus, the addition of another layer with strong mechanical characteristics like SF makes sense. The electrospun SF was immersed in ethanol to induce the β-sheet formation and become insoluble in aqueous media [17]. Then, the mixed solution of CS/PVA and CM11 peptide was electrospun on ethanol-treated SF. The final product was crosslinked using GTA vapor to become insoluble in water and form a solid bond between layers [40].

FTIR data reported from previous studies revealed that SF shows characteristic vibration peaks related to the amides group at around 1650 cm^−1^ (amide I), 1525–1540 cm^−1^ (amide II) and 1266 cm^−1^ (amide III) for α-helix motif, around 1620 cm^−^^1^ (amide I), 1514 cm^−1^ (amide II) and 1230 cm^−1^ (amide III), to β-sheet structure, 1655 cm^−1^ (amide I), 1540 cm^−1^ (amide II) and 1235 cm^−1^ (amide III) representing random coil structure [41, 42]. Similar to these reports, findings in the current study demonstrating the silk I structure of a pure SF.

The morphology of electrospun CS and SF fibers (after immersing in ethanol) reveals a highly porous and uniformed structure with interconnected networks under SEM. This structure is appropriate for a scaffold implanted in the wound bed for cell migration and proliferation and nutrients entry. The structure along with the mechanical properties of a scaffold is directly related to its potential clinical applications [43]. There are various reports in the literature regarding the addition of biodegradable biomaterials to improve the mechanical characteristics of CS scaffolds [44–46]. The addition of SF fibers has significantly enhanced these properties. According to Xu et al. [47], these characteristics are due to the content, distribution, and adhesion of the fibers. However, these improvements are most likely related to the hydrogen bonding of SF’s amide groups with N-H of CS [48].

Our biodegradation results demonstrated that the addition of SF fibers to electrospun CS effectively decreased the rate of biodegradation. In general, biodegradation of a scaffold is an essential determinant of its competence for tissue engineering approaches especially when acts as an agent carrier. A fast degradation causes a fast release of drugs and also, limits the chance of cell migration and proliferation critical for tissue regeneration. This parameter is highly related to the swelling ratio of the CS and SF alone and in combination. The swelling ratio itself displays the ability of a scaffold to absorb surrounding exudates and provide an aqueous medium required for cell growth [47, 48]. Despite the decrease in swelling rate with the addition of SF fibers compared to CS, the CS/SF scaffold still possesses around 85% rate which is appropriate for its functions in skin wound healing applications. This decrease in water uptake is due to the fact that the hydrophobic structure of SF reduces the affinity of SF-containing scaffolds with water molecules [47]. The prolonged biodegradation rate of the CS/SF scaffold facilitates the new tissue formation and controlled release of drugs [49]. The release assessment of the CM11 peptide with different concentrations from the bilayer scaffold revealed around 90% sustainable release in 8 consecutive days in all samples. A 30–40% initial release was observed in the experimental samples. This burst release on the first day may be the result of weak physical interactions of the AMP with the bilayer. Sustained release of the peptides and drugs will decrease their potential side effects and increases their effectiveness [50].

The interactions of Hu02 human foreskin fibroblasts with peptide-loaded CS/SF scaffold exhibited a well-attached and growth of cells on its surface confirmed by SEM. MTT assay was also performed to analyze the viability of these cells in contact with different concentrations of the CM11 loaded on bilayers. The results demonstrated cytotoxicity effects on the viability of cells co-cultured with CS/SF bilayer containing 64 μg/ml peptide on days 3 and 7. Therefore, this scaffold was excluded from further analysis. However, no cytotoxicity was detected in scaffolds with fewer concentrations of the peptide in 7 days. These findings are in concordance with our previous study that emphasized the dose-dependent viability of Hela, CHO, and LNCAP cells in contact with CM11 peptide [33, 50].

Disk diffusion assay was performed to evaluate the peptide-loaded bilayers’ antibacterial effects on a broad spectrum of bacterial strains. Our previous studies demonstrated the antibacterial activity of CM11 peptide against MDR strains of a wide range of pathogenic bacteria including *Staphylococcus aureus*, *Pseudomonas aeruginosa*, *Vibrio cholerae*, *Acinetobacter baumannii*, *Escherichia coli*, *Klebsiella pneumonia*, *Brucella melitensis*, and *Salmonella typhimurium* [19, 24, 33, 37]. Accordingly, the results of this study exhibited antibacterial activity of CM11 peptide-loaded CS/SF bilayer against standard strains of *S. aureus*, *E. coli, P. aeruginosa* along with their MDR strains isolated from burn patients in a dose-dependent manner. Peptide-loaded scaffolds showed a growth inhibition zone around themselves in the cultivation of standard strains, while no zone around the CS/SF disc without peptide was observed. However, the diameter size of the zones was significantly different between peptide-loaded CS/SF disks with 16 and 32 µg/ml concentrations, so that, the diameter of the zone was higher around the disk contained 32 µg/ml of the peptide. But, in MDR strains, a growth inhibition zone was observed only around peptide-loaded CS/SF disk with 32 µg/ml peptide. It worth to be mentioned that in disk diffusion assay the plates should not be incubated for more than 24 h, as this may generate invalid outcomes [28, 51]. According to this and results obtained from in vitro release profile, it demands further investigations for possible bacterial resistance to CM11 in vivo.

In general, electrospun fiber dressings are one potential technology that has been significantly investigated in recent years for wound dressing applications. They have significant advantages that make them ideal for use as wound healing dressings because they provide potential advantages in terms of increased surface area that creates a physical barrier to bacterial invasion and biofilm formation but they still retain the oxygen and gas transfer ability necessary for wound healing [52]. Similar to the current study, in a study by Song et al., an antimicrobial peptide-immobilized silk fibroin nanofiber membrane was evaluated for wound dressing application. For this purpose, an antimicrobial peptide (KR12: KRIVKRIKKWLR) originating from human cathelicidin peptide (LL37) was immobilized onto electrospun SF nanofiber membranes. Their findings confirmed antimicrobial activity against standard strains of *Staphylococcus aureus*, *Staphylococcus epidermidis*, *Escherichia coli*, and *Pseudomonas aeruginosa*. Similar to the CM11 peptide, the MIC of KR12 peptide was 8 µg/ml but they used 50, 100, 200, and 500 µg/ml concentrations of this peptide for fabrication of peptide-conjugated SF nanofiber membrane. According to the results, at a concentration of 200 µg/ml, bacterial growth was significantly inhibited. This membrane also facilitated the proliferation of keratinocytes and fibroblasts and promoted the enhanced cell–cell attachment [53]. However, the antimicrobial activity of these membranes against antibiotic-resistant strains has not been evaluated. Also, Chouhan et al., evaluated LL37-loaded PVA-silk blended nanofibrous mats as dressing application for diabetic wound healing. For this purpose, the LL37 antimicrobial peptide was loaded at 50 µg/ml concentration in nanofiber and used against standard strains of *Staphylococcus epidermidis*, and *Pseudomonas aeruginosa*. The antibacterial property of LL-37 functionalized mats was observed under both in vitro and in vivo conditions. According to their results, functionalized mats efficiently killed skin infecting *P. aeruginosa* and *S. epidermidis* bacteria. Compared to the present study, they used a peptide with a higher number of amino acids (37 aa) as well as at a higher concentration which can be increased costs [54]. On the other hand, Cai et al., fabricated a CS/SF scaffold and evaluated its antimicrobial activity alone based on the antimicrobial property of the CS against standard strains of *E*. coli and *S. aureus*. The results showed that the composite CS/SF membranes had only an antibacterial effect on *E. coli*, which its antibacterial effect increased with the increase of chitosan content. Their results demonstrated that the chitosan content is the main element of the composite which has the antibacterial effect, and its activity is varied on types of bacteria. In comparison with this study, we showed that the addition of peptide can limit the growth of MDR Gram-positive and -negative bacteria [40]. In another study conducted by Xu et al., [47], they examined the wound healing effects of chitosan/silk microfiber membrane on a full-thickness skin wound of rats. According to their findings, the addition of SF microfibers can increase the efficiency of wound healing compared to CS membrane alone. Therefore, the potential of CS/SF scaffold on infected skin wounds regeneration and repair can be perceived from the results of previous and present studies.

## Conclusion

The current study represents the antibacterial activity of an AMP-loaded CS/SF electrospun bilayer scaffold proposed for infected wound healing applications, especially for infections by MDR bacterial strains. Obtained results suggest the potential of peptide-loaded CS/FS bilayer containing 32 μg/ml of the CM11 peptide as a candidate dressing for infected wounds with MDR bacteria without cytotoxicity. We strongly believe that fabricated scaffold can be considered for in vivo studies of not only infected skin wounds but for all types of all tissues. Eventually, it looks promising as a candidate for clinical investigations.

## Supplementary information

Supplementary Materials
