# Supplementary material for: Fabrication and characterization of an antibacterial chitosan/silk fibroin electrospun nanofiber loaded with a cationic peptide for wound-dressing application
Source: J Mater Sci Mater Med. 2021 Aug 28;32(9):114. doi: 10.1007/s10856-021-06542-6 (PMC8403119; doi:10.1007/s10856-021-06542-6)
Supplement: Supplementary file 1 — Supplementary Materials [file 10856_2021_6542_MOESM1_ESM.pdf]

Gmail **Mehrdad Moosazadeh Moghaddam <mm.genetics@gmail.com>**

Article processing charge for manuscript submitted to Journal of Materials Science: Materials in Medicine

1 message

SpringerOpen Accounts <payments@springeropen.com> Fri, Jan 8, 2021 at 9:30 AM
To: Mehrdad Moosazadeh Moghaddam <mm.genetics@gmail.com>

MS: 8501238252039044
Fabrication and Characterization of an Antibacterial Chitosan/Silk Fibroin Electrospun Nanofiber Loaded with a Cationic Peptide for Wound-dressing Application
Mehrdad Moosazadeh Moghaddam,Sadjad Khosravimelal,Milad Chizari,Behrous Farhadihosseinabadi and Mazaher Gholipourmalekabadi
Journal of Materials Science: Materials in Medicine

Dear Dr Moosazadeh Moghaddam

Thank you for agreeing the article processing charge for Journal of Materials Science: Materials in Medicine. I would like to update you regarding your status with respect to the article processing charge that is normally due if a manuscript is accepted.

I am pleased to say that a waiver has been granted on this occasion.

No payment is due on publication of this article.

The article processing charge has been waived by Springer Open.

Kind regards,

BioMed Central Limited
The Campus
4 Crinan Street
London N1 9XW
UK
Phone: +44 (0) 20 3192 2009
Email: ORSupport@springernature.com
